# Supplementary material for: Modeling glioblastoma heterogeneity as a dynamic network of cell states
Source: Mol Syst Biol. 2021 Sep 16;17(9):e10105. doi: 10.15252/msb.202010105 (PMC8444284; doi:10.15252/msb.202010105)
Supplement: Supplementary file 6 — Source Data for Figure 5 [file MSB-17-e10105-s004.zip › Figure5A_sourcedata/GSEA_3017/nosigdb_stateE.GseaPreranked.1622795031093/RICHARDS_NOWAKOWSKI_SCIENCE_2017_MGE_RG1_UPREG.html]

Details for gene set RICHARDS\_NOWAKOWSKI\_SCIENCE\_2017\_MGE\_RG1\_UPREG[GSEA]

|  || Dataset | state63017 |
| Phenotype | NoPhenotypeAvailable |
| Upregulated in class | na\_pos |
| GeneSet | RICHARDS\_NOWAKOWSKI\_SCIENCE\_2017\_MGE\_RG1\_UPREG |
| Enrichment Score (ES) | 0.557403 |
| Normalized Enrichment Score (NES) | 3.5819137 |
| Nominal p-value | 0.0 |
| FDR q-value | 0.0 |
| FWER p-Value | 0.0 |
Table: GSEA Results Summary

  

Fig 1: Enrichment plot: RICHARDS\_NOWAKOWSKI\_SCIENCE\_2017\_MGE\_RG1\_UPREG      
 Profile of the Running ES Score & Positions of GeneSet Members on the Rank Ordered List

  

| PROBE | GENE SYMBOL | GENE\_TITLE | RANK IN GENE LIST | RANK METRIC SCORE | RUNNING ES | CORE ENRICHMENT || 1 | CYR61 |  |  | 3 | 1.169 | 0.0510 | Yes |
| 2 | IGFBP5 |  |  | 4 | 1.015 | 0.0981 | Yes |
| 3 | LYPD1 |  |  | 13 | 0.813 | 0.1269 | Yes |
| 4 | ANXA2 |  |  | 18 | 0.733 | 0.1565 | Yes |
| 5 | TTYH1 |  |  | 19 | 0.731 | 0.1905 | Yes |
| 6 | CNN3 |  |  | 24 | 0.702 | 0.2186 | Yes |
| 7 | IFITM3 |  |  | 26 | 0.682 | 0.2492 | Yes |
| 8 | CDH2 |  |  | 27 | 0.668 | 0.2802 | Yes |
| 9 | RAB31 |  |  | 35 | 0.598 | 0.3002 | Yes |
| 10 | HMGCS1 |  |  | 37 | 0.593 | 0.3267 | Yes |
| 11 | GPC1 |  |  | 50 | 0.530 | 0.3379 | Yes |
| 12 | MRC2 |  |  | 56 | 0.505 | 0.3558 | Yes |
| 13 | WLS |  |  | 65 | 0.472 | 0.3689 | Yes |
| 14 | SLC1A3 |  |  | 66 | 0.472 | 0.3908 | Yes |
| 15 | PDPN |  |  | 79 | 0.438 | 0.3978 | Yes |
| 16 | NES |  |  | 92 | 0.414 | 0.4036 | Yes |
| 17 | GPM6B |  |  | 105 | 0.391 | 0.4084 | Yes |
| 18 | CD99 |  |  | 107 | 0.389 | 0.4254 | Yes |
| 19 | CLU |  |  | 119 | 0.364 | 0.4301 | Yes |
| 20 | AHNAK |  |  | 125 | 0.351 | 0.4408 | Yes |
| 21 | CETN2 |  |  | 145 | 0.338 | 0.4354 | Yes |
| 22 | NEK6 |  |  | 147 | 0.337 | 0.4499 | Yes |
| 23 | GATM |  |  | 149 | 0.335 | 0.4644 | Yes |
| 24 | ZFP36L2 |  |  | 162 | 0.324 | 0.4661 | Yes |
| 25 | SPATS2L |  |  | 163 | 0.323 | 0.4811 | Yes |
| 26 | FILIP1L |  |  | 167 | 0.320 | 0.4926 | Yes |
| 27 | DDAH1 |  |  | 170 | 0.317 | 0.5051 | Yes |
| 28 | MBNL2 |  |  | 173 | 0.316 | 0.5176 | Yes |
| 29 | SLC35F1 |  |  | 183 | 0.309 | 0.5219 | Yes |
| 30 | GLUL |  |  | 196 | 0.300 | 0.5225 | Yes |
| 31 | MEST |  |  | 215 | 0.285 | 0.5157 | Yes |
| 32 | SAMD4A |  |  | 224 | 0.281 | 0.5199 | Yes |
| 33 | NPAS3 |  |  | 227 | 0.280 | 0.5306 | Yes |
| 34 | CDK6 |  |  | 238 | 0.269 | 0.5320 | Yes |
| 35 | ITGB8 |  |  | 239 | 0.268 | 0.5445 | Yes |
| 36 | QKI |  |  | 241 | 0.267 | 0.5558 | Yes |
| 37 | ATP1B2 |  |  | 260 | 0.258 | 0.5477 | Yes |
| 38 | WWTR1 |  |  | 263 | 0.256 | 0.5574 | Yes |
| 39 | ZFP36L1 |  |  | 287 | -0.255 | 0.5437 | No |
| 40 | AHI1 |  |  | 310 | -0.261 | 0.5313 | No |
| 41 | SEPT10 |  |  | 431 | -0.293 | 0.4114 | No |
| 42 | BCHE |  |  | 471 | -0.305 | 0.3822 | No |
| 43 | MCM3 |  |  | 560 | -0.340 | 0.3001 | No |
| 44 | DNER |  |  | 640 | -0.382 | 0.2300 | No |
| 45 | EGR1 |  |  | 656 | -0.398 | 0.2318 | No |
| 46 | MNS1 |  |  | 666 | -0.408 | 0.2407 | No |
| 47 | SLC25A5 |  |  | 695 | -0.426 | 0.2294 | No |
| 48 | HIST1H1E |  |  | 925 | -1.049 | 0.0234 | No |
Table: GSEA details [plain text format]

  

Fig 2: RICHARDS\_NOWAKOWSKI\_SCIENCE\_2017\_MGE\_RG1\_UPREG: Random ES distribution      
 Gene set null distribution of ES for **RICHARDS\_NOWAKOWSKI\_SCIENCE\_2017\_MGE\_RG1\_UPREG**
